# Human Penile Ossification: A Rare Cause of Sexual Dysfunction – A Case Report and Review of the Literature

**DOI:** 10.7759/cureus.12675

**Published:** 2021-01-13

**Authors:** Alex Belshoff, Alessa Aragao, Petar Bajic, Maria M Picken, Christopher Gonzalez

**Affiliations:** 1 Urology, Loyola University Medical Center, Maywood, USA; 2 Department of Pathology and Laboratory Medicine, Loyola University Medical Center, Maywood, USA; 3 Urology, Cleveland Clinic, Cleveland, USA

**Keywords:** penile ossification, sexual dysfunction, peyronie's disease

## Abstract

Human penile ossification is a rare urologic condition with approximately 40 cases reported in the literature so far. While bone is essential for penetrative intercourse in many non-human mammals, human penile ossification appears to be part of a metaplastic process occurring after injury or trauma. Conditions such as Peyronie’s disease, diabetes mellitus, local trauma, and end-stage renal disease have been associated with this entity. We report the case of a 65-year-old male with penile curvature and a history of painful intercourse who underwent partial excision and grafting with bovine pericardial graft and was found on pathologic examination to have penile ossification.

## Introduction

Human penile ossification is a rare urologic condition with approximately 40 pathologically confirmed cases reported in the medical literature to date [1-8]. While this is typically an acquired condition, there has been one documented congenital case, involving a five-year-old boy with other genitourinary defects [1]. Pathologic calcification occurs via heterotopic mineralization of the penile soft tissues and has been associated with end-stage renal disease, diabetes, trauma, malignancy, and calcium dysregulation [2-4]. However, the triggering mechanism is not entirely understood. Historically, ossification of the penis was presumed to be of vestigial origin due to the presence of penile bone in other animals, which facilitates reproduction [5]. Others have challenged this hypothesis, favoring instead a primarily metaplastic process sustained by fibrosis [6-7].

The condition most commonly associated with penile ossification is Peyronie’s disease. This condition is characterized by the development of fibrous penile scar tissue, resulting in curvature and painful intercourse, which may preclude sexual function. Surgical reconstruction is often necessary to restore sexual function. These surgical procedures include penile straightening through the use of tunica albuginea plication sutures, or partial excision of the scar with grafting into the tunical defect, among others [9]. Significant penile ossification in this setting represents a unique challenge for the reconstructive urologist [8]. In this report, we describe a unique case of Peyronie's disease with extensive corporal ossification that was managed surgically with partial excision and grafting.

## Case presentation

**Figure 1:**
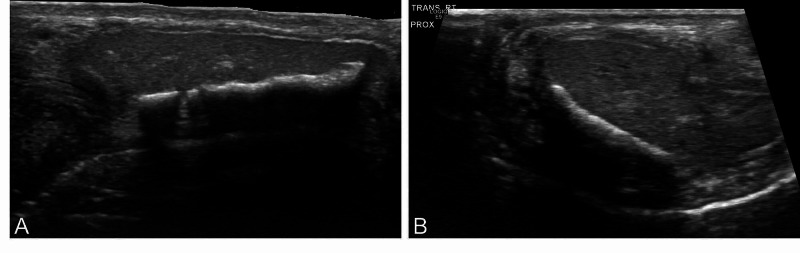
Penile Doppler ultrasound imaging demonstrating a 2.5-cm dorsal density with acoustic shadowing A) Longitudinal view of the right corpora. B) Transverse view primarily of the right corpora

A 65-year-old man was referred to our urology department for an eight-year history of dorsal curvature of the penis with erections. He described a near 90-degree curvature, which had been stable for several years and was refractory to in-office verapamil injections. He had no difficulty obtaining erections; however, they were painful and bothersome. Sexual intercourse was also difficult and painful for both him and his partner due to the curvature. His medical and surgical history was otherwise unremarkable. On exam, his penis was uncircumcised with a palpable, firm plaque along the dorsal midshaft measuring approximately 2.5 cm by 1.5 cm. A penile Doppler ultrasound was obtained, which demonstrated a broad, linear, sheet-like densification of the dorsal tunica albuginea extending from the base of the penis along most of the shaft, predominantly along the right side (Figure 1).

Due to the extensive degree of plaque and the impact on his quality of life, he was taken to the operating room for partial excision and grafting. Intraoperatively, the large, firm ossified plaque was immediately apparent, and care was taken to excise this while preserving the neurovascular bundles. The corporal defect was closed using a bovine pericardial graft (Coloplast, Minneapolis, MN). Upon intraoperative induction of an artificial erection, the partial curvature was still apparent, necessitating placement of two tunica albuginea plication sutures along the ventral shaft. Curvature was corrected to 10 degrees dorsally at the end of the procedure. The remainder of his hospital course was unremarkable, and he was discharged home the same day.

His postoperative course was also uneventful, with minimal residual curvature noted at the two-month follow-up. He denied any erectile dysfunction and is now able to obtain satisfactory erections without medications. He is sexually active with his partner and denies pain or difficulty with intercourse.

**Figure 2:**
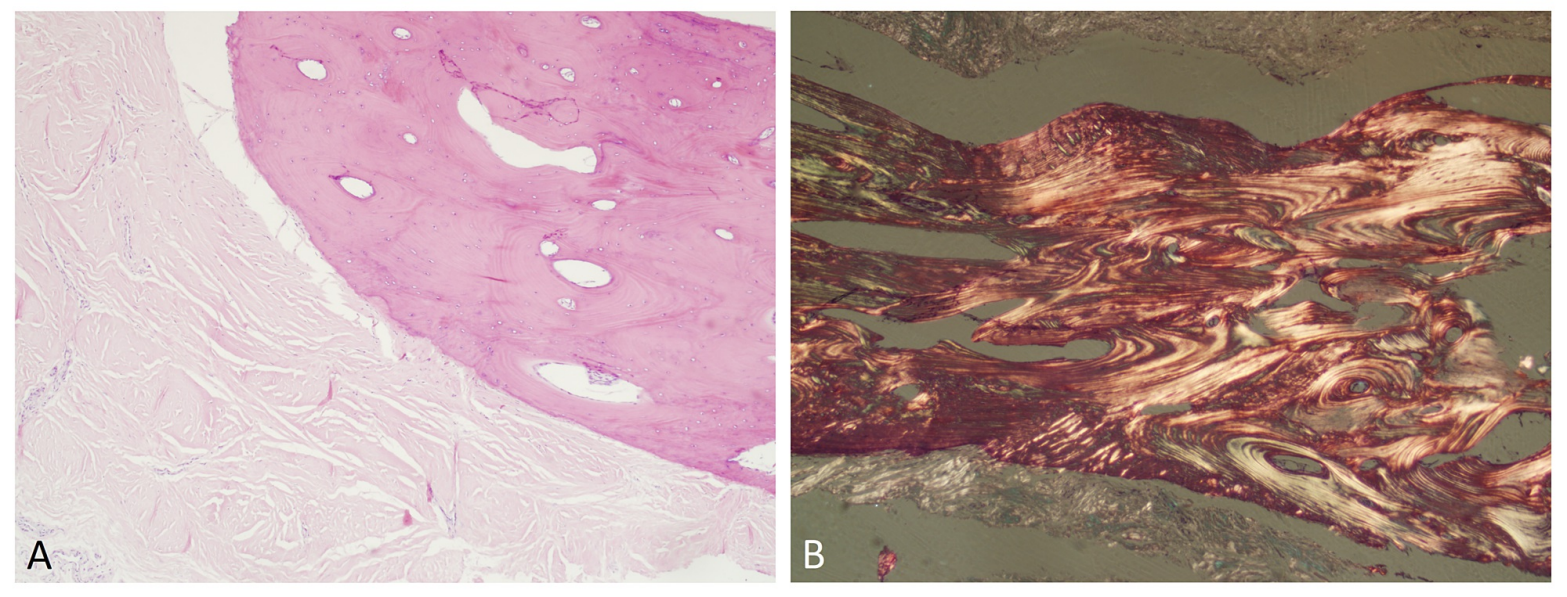
Microscopic examination A) H&E section showing bone surrounded by unremarkable stroma. B) The same section examined under polarized light showing lamellar bone. Original magnification: 300x H&E: hematoxylin and eosin

Macroscopic examination of the excised tissue revealed multiple tan-white, elongated segments of glistening tissue with central areas of calcification measuring in aggregate 3.2 cm by 1.6 cm by 1.1 cm. The tissue was examined after decalcification. The hematoxylin and eosin histologic sections demonstrated a centrally located bony tissue surrounded by penile fibrous tissue (Figure 2A). The bony tissue showed features of lamellar bone under polarized light (Figure 2B). No inflammation was present within the lamellar bone and adjacent fibrous tissue. 

## Discussion

Since the earliest case of penile ossification, reported by McClellan in 1827, only around 40 cases have been described in the literature so far. Due to the limited case count available for study, our understanding of this condition and its etiology is limited [1-8]. The presence of penile ossification often results in sexual dysfunction, necessitating referral to a urologist. This rare condition presents a unique challenge for the reconstructive surgeon as the dense, bony tissue may require excision and grafting for correction of the penile curvature. Referral to a reconstructive urologist with experience in the surgical treatment of Peyronie’s disease should be considered to optimize functional outcomes and patient satisfaction.

## Conclusions

Penile ossification is a condition that often leads to sexual dysfunction, necessitating referral to a urologist. Referral to a reconstructive urologist with experience in the surgical treatment of Peyronie’s disease should be considered to optimize functional outcomes and patient satisfaction.
